# Asymmetric activation of dimeric ATM/Tel1 kinase

**DOI:** 10.1038/s41421-025-00786-0

**Published:** 2025-03-25

**Authors:** Po Wang, Zexuan Zheng, Guangxian Wang, Zhanpeng Zhao, Dong Qian, Gang Cai, Xuejuan Wang

**Affiliations:** 1 Department of Radiation Oncology, the First Affiliated Hospital of USTC, MOE Key Laboratory for Cellular Dynamics, Division of Life Sciences and Medicine, University of Science and Technology of China, Hefei, Anhui China; 2 Key Laboratory of Anhui Province for Emerging and Reemerging Infectious Diseases, Hefei, Anhui China

**Keywords:** Cryoelectron microscopy, DNA damage response

Dear Editor,

The ataxia–telangiectasia mutated (ATM), an apical kinase that orchestrates the multifaceted DNA damage response, is the master regulator of genome stability^1^, and is a critical therapeutic target in cancer^2^. Besides being activated by DNA double-strand breaks (DSBs), ATM is also directly activated by reactive oxygen species (ROS)^3^. In 2019, we determined the cryo-EM structures of the symmetric dimer and asymmetric dimer of *Saccharomyces cerevisiae* Tel1 (the yeast homolog of human ATM) and revealed a tunable allosteric network in ATM/Tel1, which has implications for substrate recognition, recruitment, and efficient phosphorylation^4^. A recent cryo-EM structure of H_2_O_2_-activated ATM showed that under oxidizing conditions, ATM formed an intramolecular disulfide bridge inside a homodimer that promoted large-scale rearrangement of the kinase domain necessary for activation^5^. However, the activation mechanism of ATM/Tel1 in response to DNA damage remains largely unknown.

ATM/Tel1 kinase functions as an obligate homodimer with a highly dynamic N-terminal helical solenoid domain responsible for ligand binding and a conserved C-terminal FAT/kinase/FATC domain^6^. Previous cryo-EM structure of the ATM/Tel1 from *Schizosaccharomyces pombe* in the basal state has illustrated a dimer interface and the consecutive helical solenoids inhibiting the kinase activation^7^. Recently, we have improved the resolution from 7.8 Å to 4.3 Å using a new algorithm and successfully constructed a structural model (Supplementary Fig. S1, EMDB: 61024 and PDB: 9IZ7). To understand how ATM/Tel1 is activated in response to DNA damage and how the conformational rearrangements result in its activation, we purified the wild-type ATM/Tel1 in an active state bound to ATP analog and substrate peptide, and analyzed this preparation by cryo-EM.

We pre-treated the cultured cells with DSB-inducing methylmethane sulfonate and endogenously activated the cellular ATM/Tel1. The affinity-purified complex is stoichiometric, highly pure, and displays stimulated kinase activity (Supplementary Figs. S2a and S3). Moreover, the ATM/Tel1 activity could be additionally stimulated by 329-bp blunted end DNA. Based on a radiolabeled ATP-based kinase assay, which is the “gold standard” for the quantification of protein kinase activity, we managed to achieve a 30-fold increase in ATM/Tel1 activity (Fig. 1a; Supplementary Fig. S3). This level of activation is comparable with the activation of human ATM by H_2_O_2_ in vitro^5^. Furthermore, to investigate whether the activity of activated ATM/Tel1 can be further enhanced, we assembled MRN in vitro and confirmed its assembly using glycerol density gradient centrifugation (Supplementary Fig. S4a–c). We observed that MRN efficiently activates basal ATM/Tel1 and exhibits robust DNA cleavage activity (Supplementary Figs. S4d and S5). However, it does not further enhance the activity of the active ATM/Tel1 (Supplementary Fig. S5), indicating that ATM/Tel1 is already in a maximally activated state due to endogenous activation combined with DNA stimulation.

**Fig. 1:**
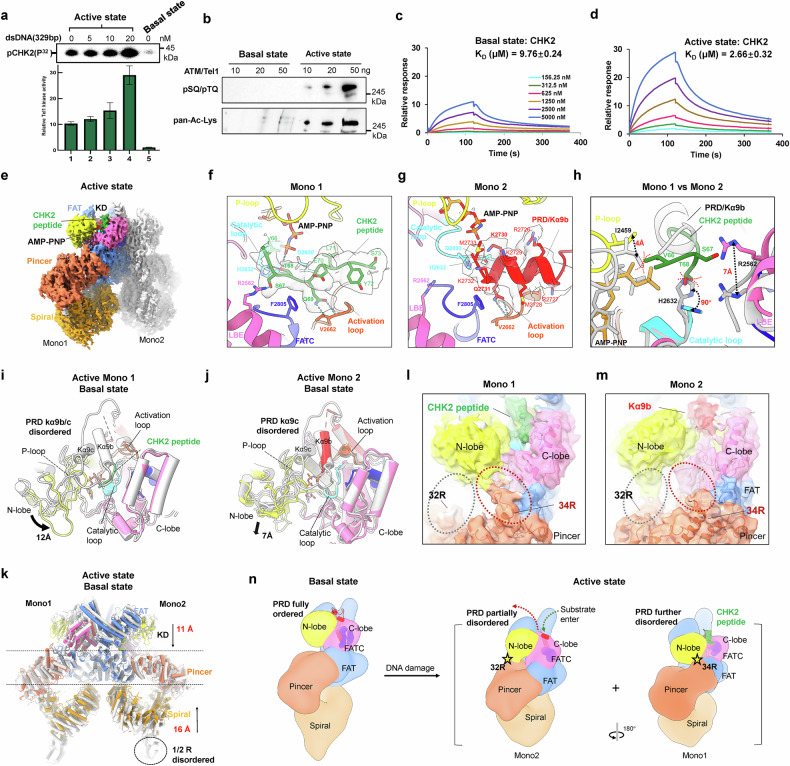
Structure of the activated ATM/Tel1 kinase bound to the ATP analog and substrate peptide. **a** A radiolabeled ATP-based kinase assay using the basal and active ATM/Tel1 (5 nM) with and without 329-bp blunted DNA. The different amounts of DNA added are labeled. Bar representations of the relative kinase activities based on the three independent phosphorimaging quantitations are shown below. **b** The purified basal and active ATM/Tel1 proteins were analyzed by western blotting using phospho-ATM/ATR substrate motif (pS/pT) Q and pan-acetylated-lysine antibodies. The amounts of ATM/Tel1 used for the analysis are indicated. **c**, **d** SPR sensorgram depicting substrate-binding affinity to basal (**c**) and active (**d**) ATM/Tel1 at varied CHK2 concentrations. **e** The cryo-EM structure of the active ATM/Tel1 dimer. Mono1 is color-coded by domain assignment: Spiral in orange, Pincer in coral, FAT in cornflower blue, kinase N-lobe in yellow, C-lobe in hot pink, PRD in red, FATC in blue, and CHK2 peptide in dark green. Mono2 is shown as a solid gray surface. **f**, **g** The structures of the active sites in both monomers. The active site in Mono1 is bound to the substrate peptide, while that in Mono2 is occupied by PRD kα9b with the interfaces denoted. **h** The conformational differences of the two monomers’ active sites in the active ATM/Tel1, highlighting two significant steric hindrances and a shift in Arg2562. The steric hindrances are depicted with red dashed lines. **i**, **j** The superimposed active site structures of active Mono1 (**i**) or active Mono2 (**j**) and the basal state, aligned on their kinase C-lobe. The disorder of the PRD and the motion of the N-lobe are highlighted. The basal-state structure is colored gray. **k** Global structural comparison of the active state (domain color-coded as in **e**) and the basal state (gray pipes), aligned on the Pincer in Mono1. **l**, **m** The structural comparison of the two active monomers, highlighting that the N-lobe alternatively docks onto the two different pliers of the Pincer. **n** The activation model of ATM/Tel1 in response to DNA damage (see the main text for details).

ATM activation generally involves autophosphorylation and acetylation modifications^6^. The activated ATM/Tel1 exhibits strong autophosphorylation and acetylation modifications, which may explain the sustained activation of ATM/Tel1 (Fig. 1b; Supplementary Fig. S6). Furthermore, compared to the basal state of ATM/Tel1, the activated ATM/Tel1 harbors a higher binding affinity towards substrate peptides (improved by ~3.7-fold), as revealed by surface plasmon resonance (SPR) (Fig. 1c, d). Surprisingly, the CHK2 peptide exhibits an affinity for active ATM/Tel1 comparable to that of the full-length CHK2. However, its affinity for basal ATM/Tel1 is reduced by over 120-fold compared to the active state (Supplementary Fig. S7), likely due to the absence of an appropriate binding interface. These biochemical and functional assays consistently suggest that ATM/Tel1 is robustly activated.

After incubating the active wild-type ATM/Tel1 with the CHK2 substrate peptide (CHK2^63–74^), and AMP-PNP in vitro, we froze the specimen and determined the cryo-EM reconstruction, which revealed that ATM/Tel1 adopts three distinct conformations (Supplementary Figs. S2b, c and S8). Two of the conformations, Structure 1 and Structure 3, which are closer to the basal and active states, respectively, do not bind to the substrate peptide. These structures were resolved at nominal global resolutions of 3.6 Å and 4.0 Å, respectively. We focused on analyzing Structure 2 of the active state that binds to the substrate peptide, with a resolution of 3.6 Å (Fig. 1e).

Unexpectedly, only one monomer in the active dimer binds to the substrate peptide. We designated the substrate-bound monomer as Mono1 and the other as Mono2. The peptide density is observed in Mono1 (^66^VSTQELYS^73^) (Fig. 1f; Supplementary Fig. S9). The hydroxyl group of the phospho-acceptor threonine (Thr68) in the peptide substrate is oriented towards the γ-phosphate of AMP-PNP, forming a hydrogen bond with His2632 in the catalytic loop. The glutamine (Gln69) sits in a hydrophobic pocket formed by the activation loop and the FATC domain, where it stacks against Phe2805 (FATC) and forms a pair of hydrogen bonds with the backbone of Val2662 (activation loop). In addition, Ser67 forms a hydrogen bond with Arg2562 in the LST8-binding element (LBE), further stabilizing the CHK2 peptide within the active site (Fig. 1f). However, the Lys2730 of the kα9b in the PIKK regulatory domain (PRD) of Mono2 occupies the Thr68-binding site, and Gln2731 occupies the Gln69-binding site, thereby competitively preventing substrate binding in the catalytic center (Fig. 1g). This is the first observation that both substrate peptide and pseudo-substrate PRD bind to the active sites of the identical ATM/Tel1 dimer molecule.

There are significant conformational differences in the active sites of the two monomers within the active ATM/Tel1–substrate complex, preventing effective substrate binding in Mono2 (Fig. 1h). Thr68 and Val66 from the CHK2 peptide bound to Mono1 clash with His2632 in the catalytic loop and Ile2459 in the P-loop of Mono2, respectively. In addition, Arg2562 in the LBE region of Mono2 deviates by ~7 Å from its position in Mono1, preventing the formation of a stabilizing hydrogen bond with Ser67 in the peptide. Together, the conformational differences result in steric hindrances and disrupt the formation of substrate-binding hydrogen bonds, thereby hindering substrate binding in Mono2.

The PRD (residues 2719–2780) is comprised of the kα9b (residues 2724–2732), kα9c helices (residues 2747–2751), and a long loop connecting them. In its basal state, the PRD features fully ordered kα9b, kα9c, and the linker loop, which collectively create a complete obstruction to substrate access to the active site (Supplementary Fig. S1b). This structural arrangement likely accounts for the significantly lower affinity of the CHK2 peptide to the basal-state ATM/Tel1 (Supplementary Fig. S7b). In the active form of Mono1, both kα9b and kα9c are disordered, enabling the substrate peptide to bind at the active site (Fig. 1i). In contrast, in the active state of Mono2, only kα9c is disordered, while kα9b remains in place, blocking access to the substrate-binding site (Fig. 1j).

Besides the PRD, there are also significant conformational differences in the kinase N-lobe, whereas the C-lobe, including the activation loop (residues 2646–2670) and the catalytic loop (residues 2628–2636), remains largely unchanged (Fig. 1i, j). In contrast with the basal state, the N-lobe in Mono1 moves 12 Å closer to the active site cleft, which narrows the active site and clamps the substrate (Fig. 1i), whereas that of Mono2 moves 7 Å downward away from the active site cleft. This conformational change widens the active center and facilitates substrate entry or product release (Fig. 1j).

Upon activation, the Spiral shifts upward towards the FAT-Pincer, and the FAT harbors a coordinated conformational change, with the height reduced from 200 Å in the basal state to 164 Å (Supplementary Fig. S10a). The Spiral moves upward towards the Pincer by ~16 Å, whereas the kinase N-lobe moves down towards the Pincer by ~11 Å (Fig. 1k; Supplementary Figs. S10 and S11). The N-terminal part of the Spiral (HEAT repeats 1 R and 2 R) is largely disordered (Fig. 1k). The extensive global changes in the activated ATM/Tel1 recapitulate the activation process of DNA-PKcs in which the local stretch and twist of HEAT repeats culminate in its activated state^8^ (Supplementary Fig. S12). These observations suggest that the conformation of the N-terminal solenoids is directly coupled to changes in the active site and transition in the PRD from ordered to disordered.

The two divergent conformations of the kinase active site within the identical activated ATM/Tel1 dimer are achieved through alternative docking of the N-lobe at two distinct docking sites on the Pincer (HEAT 32 R or 34 R; Fig. 1l, m; Supplementary Fig. S13). The Mono1 N-lobe packing against HEAT 34 R (residues 1583–1610) clamps the substrate peptide and defines a confined catalytic chamber (Fig. 1l), whereas the N-lobe docking at HEAT 32 R (residues 1470–1483) in Mono2 stabilizes a widened active center and facilitates substrate entry or product release (Fig. 1m). In Structure 3, we also observed a similar conformational change; however, unlike in Structure 2, only the disordering of kα9b was detected, without the binding of the substrate peptide (Supplementary Fig. S14). The two docking sites of the N-lobe are consistent with the observation in the endogenously activated ATR/Mec1^9^ (Supplementary Fig. S15). The proper alignment of the N- and C-lobes is central to kinase activation^8,10,11^. These observations illuminate that the Pincer in active ATM/Tel1 and ATR/Mec1 enables the realignment of the two kinase lobes and facilitates kinase activation.

Recently, the structure of H_2_O_2_-activated ATM bound to a p53 peptide was determined^5^. The ATM in this sample contains a mutated residue in the PRD (Q2971A), and showed enhanced substrate phosphorylation under both basal and H_2_O_2_-activated conditions. ATM activated by ROS exhibits structural symmetry with both monomers bound to the substrate peptide. The two monomers are rotated relative to each other, instead of the global compression observed in the active wild-type ATM/Tel1 in this study, which is accompanied by the release of the substrate-blocking PRD region (Supplementary Fig. S16). Furthermore, the asymmetric active ATM/Tel1 exhibits an expanded catalytic environment due to the docking of the N-lobe onto the Pincer. Conversely, in the H_2_O_2_-activated ATM, the formation of disulfide bonds and the rotation between monomers cause the P-loop and nucleotide-binding region to shift closer to the dimer interface (Supplementary Fig. S17). Moreover, we compared the active site of active ATM/Tel1 with those of other published activated PIKK family members. By aligning the activation loop and catalytic loop as references, we observed similar conformational changes in the P-loop: the nucleotide-binding pocket moves toward the catalytic loop and activation loop^8,10,12^ (Supplementary Fig. S18). We postulate that these asymmetric and compressive rearrangements observed in the active ATM/Tel1 are crucial for DNA damage-induced activation.

We are intrigued by why only one monomer in the ATM/Tel1 dimer binds to a substrate peptide. To verify whether both monomers could be activated simultaneously, we incubated active ATM/Tel1 with over-saturated full-length CHK2 and fractionated the assembled complex using GraFix^13^. Mass photometry analysis was used to determine the molecular weight and stoichiometry of the complex, revealing that only one CHK2 molecule is bound to an ATM/Tel1 dimer (Supplementary Fig. S19). Structural analysis also confirmed that only Mono1, but not Mono2, can bind to the CHK2 peptide. In the active Mono1, kα9b becomes disordered and disrupts the PRD–LID’ (LBE-interacting domain) interface, creating space for the substrate to enter the active site. The PRD’–LID interface in Mono2 is retained to maintain the ATM/Tel1 dimer structure, and the ordered PRD’ blocks substrate entry (Supplementary Fig. S20). Together, the asymmetric structures of the active sites in two monomers and the binding of the substrate to only one monomer in the dimer are likely mechanisms underlying ATM/Tel1 dimer activation.

We show that ATM/Tel1 in the basal state adopts a symmetric dimer configuration with the kinase N-lobe suspended, keeping the N- and C-lobes misaligned, and the PRD fully ordered, blocking the substrate access to the active site (Supplementary Fig. S1). Upon activation, ATM/Tel1 undergoes global compressive conformational changes and transforms into an asymmetric dimer, where only one monomer in the ATM/Tel1 dimer binds to a substrate peptide. When the N-lobe docks on 34 R, the PRD is fully disordered, and the substrate binds in a sealed catalytic chamber. When the N-lobe docks on 32 R, the PRD partially occupies the active center, facilitating substrate entry or product release. The N-lobe toggles between the two alternative stable docking sites and recurrently commits the two monomers for iterative substrate recruitment, confined catalysis, and product release (Fig. 1n).

In short, this study provides the first structure of DNA damage-activated ATM/Tel1 bound to a protein substrate, which illuminates the asymmetric activation mechanism of the dimeric ATM/Tel1 kinase. Targeting the asymmetric activation features may facilitate the development of more potent ATM inhibitors for cancer therapy.

## Supplementary information

Supplementary Information

## Data Availability

Cryo-EM maps have been deposited in the Electron Microscopy Data Bank (EMDB) under accession numbers EMD-61024 (basal state), EMD-61006 (active state with substrate peptide bound), EMD-61018 (basal state with Spiral and PRD partially disordered), EMD-61014 (active state without substrate peptide bound). Model coordinates have been deposited in the Protein Data Bank (PDB) under accession numbers 9IZ7 (basal state), and 9IZ0 (active state with substrate peptide bound).
