# Supplementary material for: Asymmetric activation of dimeric ATM/Tel1 kinase
Source: Cell Discov. 2025 Mar 25;11:30. doi: 10.1038/s41421-025-00786-0 (PMC11933327; doi:10.1038/s41421-025-00786-0)
Supplement: Supplementary file 1 — Supplementary Information [file 41421_2025_786_MOESM1_ESM.pdf]

Supplementary Information

Mechanisms of ATM/Tel1 kinase Activation in Response to DNA Damage

Authors: Po Wang [1], Zexuan Zheng [1], Guangxian Wang [1], Zhanpeng Zhao [1], Dong Qian [1], Gang Cai [1,2]*, Xuejuan Wang [1]*

Affiliations:

[1] Department of Radiation Oncology, the First Affiliated Hospital of USTC, MOE Key Laboratory for Cellular Dynamics, Division of Life Sciences and Medicine, University of Science and Technology of China, Hefei, China 230027.

[2] Key Laboratory of Anhui Province for Emerging and Reemerging Infectious Diseases, Hefei, Anhui, China.

* Correspondence: Xuejuan Wang (xuejuan@ustc.edu.cn) and Gang Cai (gcai@ustc.edu.cn)

Purification of Yeast ATM/Tel1

S. pombe stain CC5060 (*h–leu1-32 ura4-D18 kanMX6:nmt1:TAP-tel1*)[1] was grown in YPD medium to the stationary phase. 0.05% MMS（MACKLIN）was added at OD600 ≈ 4 and cells were collected 60 min later. About 100 g cells were harvested, washed, and resuspended in extraction buffer (50 mM HEPES [pH 7.6], 300 mM ammonium sulfate, 0.5 mM EDTA, 5 mM β-ME, 10% (v/v) glycerol, 0.01% (v/v) NP-40 and protease inhibitors), and a whole-cell extraction was prepared as previously described[2]. This whole-cell extract was selectively precipitated in 30-55% ammonium sulfate and resuspended using binding buffer (50 mM HEPES [pH 7.6], 1 mM EDTA, 10 mM $ZnCl_2$, 5 mM β-ME, and protease inhibitors). After the suspension was clarified using centrifugation, the supernatant was incubated for 2 h at 4 °C with 1 ml of a 50% slurry of IgG-sepharose resin beads (GE Healthcare) that had been pre-equilibrated with binding buffer plus 250 mM ammonium sulfate. After incubation, the beads were washed with wash I buffer (50 mM HEPES [pH 7.6], 500 mM ammonium sulfate, 0.5 mM EDTA, 5 mM β-ME, 10% (v/v) glycerol, 0.01% (v/v) NP-40 and protease inhibitors), followed by a second wash with 50 ml of wash II buffer (50 mM HEPES [pH 7.6], 50 mM ammonium sulfate, 0.5 mM EDTA, 5 mM β-ME, 10% (v/v) glycerol, 0.01% (v/v) NP-40 and protease inhibitors). After equilibration of the column with digestion buffer (50 mM HEPES [pH 7.6], 100 mM ammonium sulfate, 0.5 mM EDTA, 5 mM β-ME, 10% (v/v) glycerol and 0.01% (v/v) NP-40), 100 units of Ac TEV protease (NEB) was added to the resin beads and incubated overnight at 4 °C. The ATM/Tel1 fraction was then eluted with three column volumes of digestion buffer. The ATM/Tel1 elution was flash-frozen in liquid nitrogen, and analyzed by SDS-PAGE and EM examination.

Expression and Purification of full-length CHK2, MRN (Mre11, Rad50-core, Nbs1)

The codon-optimized gene fragments for human full-length CHK2, yeast Mre11, Rad50-core (lacking the coiled-coil motif) were cloned into the pET22b vector with a C-terminal 6× His tag and Nbs1 were cloned into the pET22b vector with a C-terminal 6× His tag and a GST tag, and transformed into *E. coli* BL21(DE3). Cells were cultured in 2 liters of LB medium at 37°C and 220 rpm until reaching an OD600 of 0.6. Protein expression was induced by adding 0.5 mM IPTG, followed by incubation for 12 hours at 16°C and 220 rpm. Cells were harvested by centrifugation at 8,000g for 10 minutes at 4°C. The cell pellet from 2 liters of culture was resuspended in digestion buffer containing 10 mM imidazole. Cells were lysed by sonication at 4°C, with a total sonication time of 10 minutes (3-second pulses with 3-second intervals). The lysate was clarified by centrifugation at 16,000g for 30 minutes at 4°C. The supernatant was incubated with 1 ml of Ni-NTA beads (Smart-Lifesciences) for 1 hour at 4°C. Following incubation, the beads were washed with digestion buffer containing 50 mM imidazole. After washing, 1 ml of digestion buffer containing 50 mM imidazole was added to the beads and incubated for 1 hour at 4°C. Protein was then eluted with three-column volumes of elution buffer. The eluted CHK2 and MRN proteins were flash-frozen in liquid nitrogen and analyzed by SDS-PAGE and EM.

Preparation of MRN (Mre11, Rad50-core, Nbs1) complex

To assemble the MRN complex, 100 μl of Mre11 at 0.1 μM was combined with 100 μl of Rad50-core at 0.1 μM and 50 μl of Nbs1 at 0.1 μM in a buffer containing 50 mM HEPES (pH 7.6), 100 mM ammonium sulfate, 0.5 mM EDTA, 5 mM β-ME, 10% (v/v) glycerol and 0.01% (v/v) NP-40. The mixture was incubated at 4 °C for 60 minutes and the reaction mixture was then applied to the

top of a 4-ml glycerol gradient (10–20%) in a buffer with 50 mM HEPES (pH 7.6), 100 mM ammonium sulfate, 0.5 mM EDTA, 5 mM β-ME, and 0.01% (v/v) NP-40 for glycerol gradient centrifugation. The gradient was centrifuged using a Beckman SWI60 rotor in a Beckman Optima ultracentrifuge at 40,000 rpm for 18 hours at 4 °C. Fractions were collected and analyzed by SDS-PAGE and EM.

Substrate peptide

The 12-residue CHK2 substrate peptide (human CHK2 peptide 63-LETVSTQELYSI-74) was custom-synthesized by Sangon, China, with an N-terminal NH2 and C-terminal COOH, in phosphate salt form. The lyophilized powder was dissolved to 1mg/mL in buffer containing 50 mM Hepes (pH 7.5), 100 mM ammonium sulfate, and 10% glycerol, aliquoted, flash-frozen, and stored at −80°C.

Preparation of DNA

The 329 bp PCR products were amplified from the plasmids containing the ATM/Tel1 gene. The 329 bp PCR products were gel-purified. To make DNA duplexes, oligos were annealed in 20 mM Tris-HCl (pH 8.0), 50 mM NaCl, and 0.5 mM EDTA buffer in a Thermocycler by heating to 95 ℃ for 5 min and cooling to 4 ℃ over 1 hour. The DNA sequences used in this study are listed in Supplementary Table S2.

DNA resection assay

The standard assay (10 µL) contained 35 nM MRN complex and 1 µM of various DNA substrates in a reaction buffer consisting of 50 mM HEPES (pH 7.6), 100 mM ammonium sulfate, 10% (v/v) glycerol, 10 µM $ZnCl_2$, 5 mM β-mercaptoethanol (β-ME), 8 mM magnesium acetate, and 0.1 mM ATP. The reactions were incubated at 30 °C for 60 minutes and terminated by mixing with an equal volume of 2% SDS/25 mM EDTA. Subsequently, the samples were adjusted to a final volume of 15 µL with buffer and treated with 1 µL of proteinase K (10 mg/mL) at 37 °C for 2 hours. The resection products were then analyzed by 3% TAE/agarose gel electrophoresis.

Kinase activity assay

We performed an *in vitro* kinase assay using the purified human CHK2 (kinase-dead mutant, containing 1-219 residues, referred to as CHK2 below) as a representative substrate. The 10 µl standard assay contained 50 mM HEPES [pH 7.6], 100 mM ammonium sulfate, 10% (v/v) glycerol, 10 µM $ZnCl_2$, 5 mM β-ME, 8 mM magnesium acetate (final concentration, including contributions made by protein storage buffers). For the reactions with DNA or MRN, basal ATM/Tel1 and active ATM/Tel1 were first incubated with DNA or MRN at 30º C for 30 min and then the substrate and 0.1 µCi [γ-P32]-ATP were added followed by further incubation at 30 ºC for 30 min, stopped with SDS-PAGE loading dye, boiled, and separated on 4-20 % SDS-PAGE gels (Keygen BioTECH, Cat. NO: KGC4716-2). The gels were dried and exposed to a phosphor screen (GE Healthcare). Data quantification was carried out using ImageQuant software.

Western blot

To detect ATM/Tel1 acetylation and autophosphorylation *in vitro*, we endogenously purified basal and active ATM/Tel1. Different amounts of purified basal and active ATM/Tel1 were then subjected

to SDS-PAGE and analyzed by Western blot (WB) using pan-acetylated-Lysine (Rabbit monoclonal, Cat# 9441S, Cell Signaling Technology). and phospho-ATM/ATR substrate (pSQ/pTQ motif, Rabbit monoclonal, Cat# 6966, Cell Signaling Technology) antibodies.

Surface plasmon resonance

Interaction analysis between ATM/Tel1 in different states and CHK2 was performed at 20 °C using a Biacore 8K (GE Healthcare) by immobilizing CHK2 onto a CM5 sensor chip. A twofold dilution series of antibodies was injected at a flow rate of 30 µl min^{-1} in PBS running buffer. All conditions were tested for at least five different antibody concentrations, and the blank channel of the chip served as the negative control. After each cycle, the sensor was regenerated with 10 mM NaOH. Data processing and kinetic analyses were performed using Biacore 8K Evaluation Software (Cytiva).

GraFix

To assemble the ATM/Tel1-CHK2 complex, 400 µl of active ATM/Tel1 at 0.1 µM was combined with 100 µl of full-length CHK2 at 4 µM in a buffer containing 50 mM HEPES (pH 7.6), 100 mM ammonium sulfate, 0.5 mM EDTA, 5 mM β-ME, 10% (v/v) glycerol, 0.01% (v/v) NP-40, 8 mM magnesium acetate, and 1 mM AMP-PNP. The mixture was incubated at 30 °C for 30 minutes, followed by an additional 30-minute incubation at 4 °C. The reaction mixture was then concentrated to a final volume of 200 µl and applied to the top of a 4-ml glycerol gradient (10–30%) containing 0.01–0.1% glutaraldehyde in a buffer with 50 mM HEPES (pH 7.6), 100 mM ammonium sulfate, 0.5 mM EDTA, 5 mM β-ME, and 0.01% (v/v) NP-40 for GraFix. The gradient was centrifuged using a Beckman SWI60 rotor in a Beckman Optima ultracentrifuge at 36,500 rpm for 16 hours at 4 °C. Fractions were collected, and the crosslinking reaction was quenched by adding ice-cold Tris-HCl (pH 8.0) to a final concentration of 50 mM.

Mass photometry

To determine the stoichiometry and mass distribution between active ATM/Tel1 and its substrate CHK2, standard mass photometry landing assays were performed at 25 °C using a TwoMP instrument (Refeyn) mounted on an active anti-vibration platform. Silicone gaskets and glass coverslips (Refeyn) were used, and coverslips were cleaned by sequential sonication in 100% (v/v) isopropanol, ultrapure water, and final plasma glow discharge. Each sample was maintained in digestion buffer (50 mM HEPES [pH 7.6], 100 mM ammonium sulfate, 0.5 mM EDTA, 5 mM β-ME, 10% (v/v) glycerol, and 0.01% (v/v) NP-40) until the moment of measurement. Full-length CHK2 was diluted 1000-fold, active ATM/Tel1 was diluted 5-fold, and the ATM/Tel1-CHK2 complex (mixture of GraFix fraction 13-17) was used without dilution in a detergent-free buffer immediately before measuring a 60–120 s image series. The final protein concentration at the time of measurement was 20 nM. Bovine serum albumin standards (Beyotime) were measured on the same day to calibrate extracted particle contrast relative to mass. Image series were acquired with an 8 ms exposure (128 Hz) at 488 nm, covering a 12 × 17 µm field of view. Frame and pixel binning were applied, with binning factors of 3 and 6, respectively, resulting in an effective pixel size of 72 nm. Data acquisition and analysis were conducted using the Refeyn AcquireMP and DiscoverMP software packages (v.2.5). To assess the impact of NP-40 in the digestion buffer, we conducted control tests on the buffer alone, which indicated that 0.01% NP-40 forms 200 kDa micelles.

EM grids preparation

The affinity chromatography sample of ATM/Tel1 was diluted to final concentrations of 0.05 mg/ml (50 mM HEPES [pH 7.6], 0.01% (v/v) NP-40, 1 mM EDTA, 10 mM $ZnCl_2$, 5 mM β-ME, and protease inhibitors). Negative staining was used to evaluate the protein quality. In brief, 3 μl of freshly purified ATM/Tel1 was applied onto copper grids supported by a thin layer of glow-discharged carbon film (Zhongjingkeyi Technology Co., Ltd). After adsorption for 30 s, uranyl acetate (2% w/v) was used for negative staining at room temperature. The negatively stained grids were examined using FEI Talos L120C operated at 120 KeV. Cryo-grid preparation was performed using EM GP2 (Leica company) operated at 4 °C and 100% humidity. Aliquots of 3 μl of sample (purified active ATM/Tel1, 329 bp DNA and human CHK2 peptide substrate were mixed at a molar ratio of 1: 20: 200 and incubated with AMPPNP and on ice for 1 h) were applied to freshly homemade GO grids. The grids were blotted using filter paper and then flash-plunged into liquid ethane pre-cooled in liquid nitrogen. All grids were screened using a Talos Glacios cryo-electron microscope (Thermo Fisher Scientific) operated at 200 keV.

Cryo-EM data collection

The grids were imaged in a Titan Krios cryo-electron microscope (Thermo Fisher Scientific) operated at 300 keV at a magnification of 81,000 × (corresponding to a calibrated sampling of 1.07 Å per pixel). Movies were recorded by EPU with a Gatan K3 Summit direct electron detector, where each movie was composed of 32 individual frames with an exposure time of 3.8 s and a dose rate of 50 electrons per second per $Å^2$. Finally, 6,333 movie stacks were collected with a defocus range of 1.5-2.5 μm by EPU software.

Data processing

Beam-induced sample motions were corrected using MotionCorr2[3] software, and contrast transfer function (CTF) parameters were estimated using CTFFIND4[4] software. All particles were autopicked using the Gautomatch (developed by Kai Zhang, http://www.mrc-lmb.cam.ac.uk/kzhang/Gautomatch/) and further checked manually. All reported resolutions were calculated from gold standard refinement procedures with the FSC = 0.143 criterion implemented in cryoSPARC[5]. For the active ATM/Tel1 dataset, after multiple rounds of 2D and 3D classifications, 378,425 particles were selected and reextracted by RELION4.0. The particles are then imported into cryoSPARC for further refinement. Using non-uniform refinement, we preliminarily obtained a 3.53 Å map with a compact conformer and then performed a masked 3D classification without alignment (soft mask: kinase domain), we obtained three different conformations, a 3.60 Å most similar to that of the basal state, except the Spiral and PRD regions partially disordered, a 3.63 Å active conformer bound to CHK2 peptide, and a 4.03 Å active conformer without substrate peptide, respectively. However, no extra DNA density was found within these three maps.

Model building

We generated a homology model of the *S. pombe* ATM/Tel1 based on *Chaetomium thermophilum* Tel1(PDB: 6SL0) [6] using SwissServer [7], and performed rigid body docking into the density of the active ATM/Tel1 using UCSF Chimera[8]. Further rigid body fitting was performed in

PHENIX[9] and residues that did not fit the density or that clearly differed between species were trimmed in Coot[10]. All structures were refined against their corresponding maps with real space refinement in PHENIX, using Ramachandran and C_β restraints and further restraints for secondary structure. Ramachandran, C_β, non-crystallographic symmetry (NCS), and secondary structure restraints (generated in PHENIX using caBLAM[11]) were used throughout the refinement to ensure good model geometry and the coordinates were validated using MOLPROBITY in PHENIX. Typical 3-cycles of real space refinement were run (3 macrocycles of global and local optimization and B-factor refinement), with PHENIX automatically estimating the relative weighting of the restraints and map to prevent over-fitting. Refinement and model statistics are given in Supplementary Table S1.

Model Interpretation and Analysis

All figures containing models and EM maps were prepared with Chimera and ChimeraX [12].

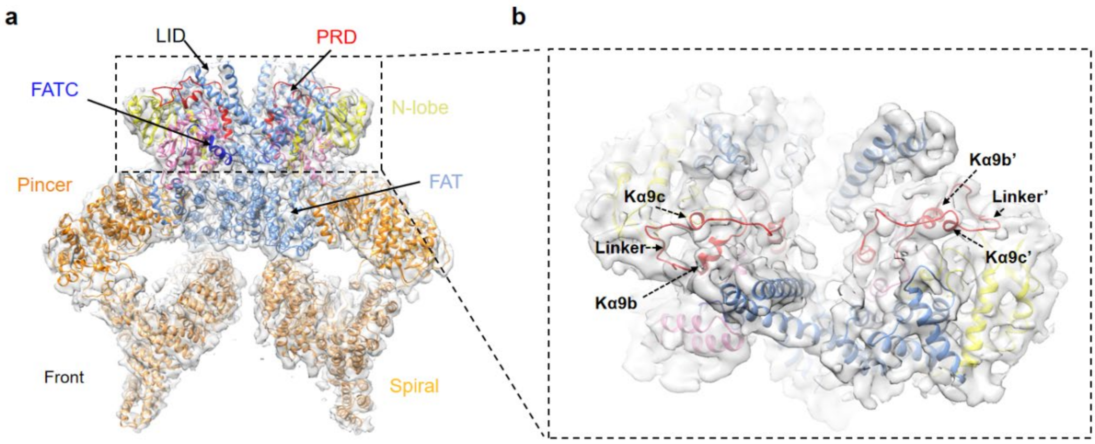

Fig. S1: The improved cryo-EM structure of ATM/Tel1 in basal state.

(a) Basal state ATM/Tel1 in a butterfly-shaped symmetric organization. The cryo-EM density is shown as a translucent surface, fitted with the ribbon diagram model color-coded as in Figure. 1e. (b, c) A close-up view of the active site highlights that the PRD' kα9b (2,724-2,732) and kα9c (2,747-2,751) occupy the active site.

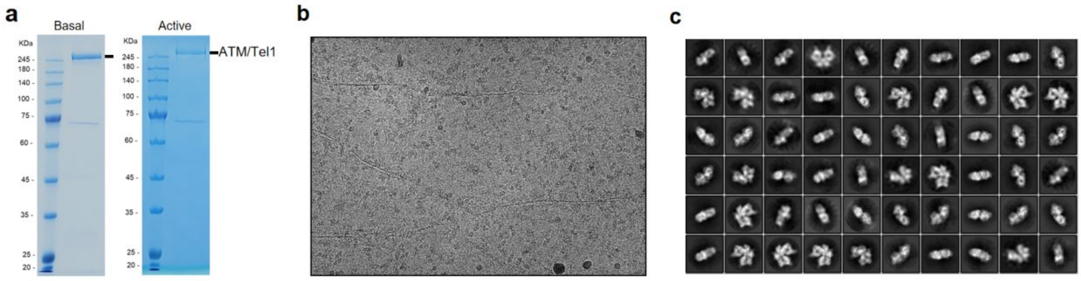

Fig. S2: Preparation of active ATM/Tel1.

(a) SDS-PAGE analysis of ATM/Tel1 endogenously purified from the fission yeast *Schizosaccharomyces pombe*. The left panel shows the purification of the basal state, and the right panel shows the purification of the active state. (b) Representative micrograph of the dataset used to determine the active ATM/Tel1 structure. (c) Representative 2D average of active ATM/Tel1.

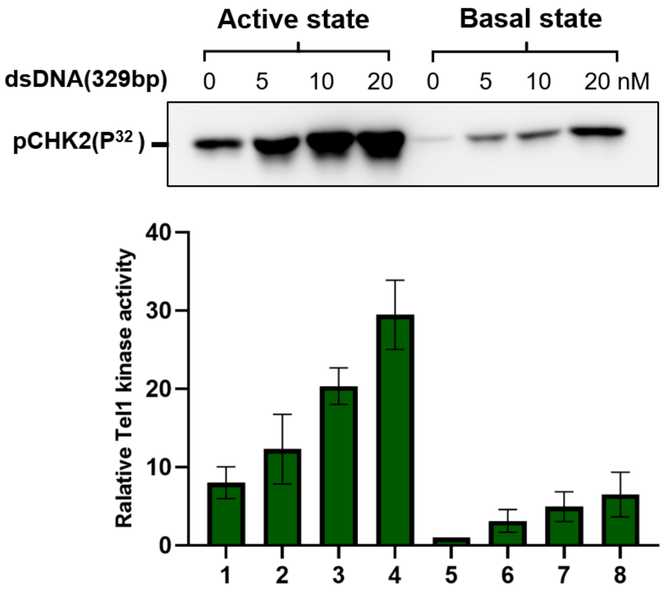

Fig. S3: DNA-induced *in vitro* activation of ATM/Tel1 in basal and active states independent of MRN.

The *in vitro* kinase assays were performed using basal and active ATM/Tel1 (5 nM) in the presence of increasing amounts of 329 bp blunt DNA fragments. The specific amounts of DNA used are indicated. Bar representations of the relative kinase activities were generated based on the phosphorimaging results. Averages and standard errors were derived from six independent experiments.

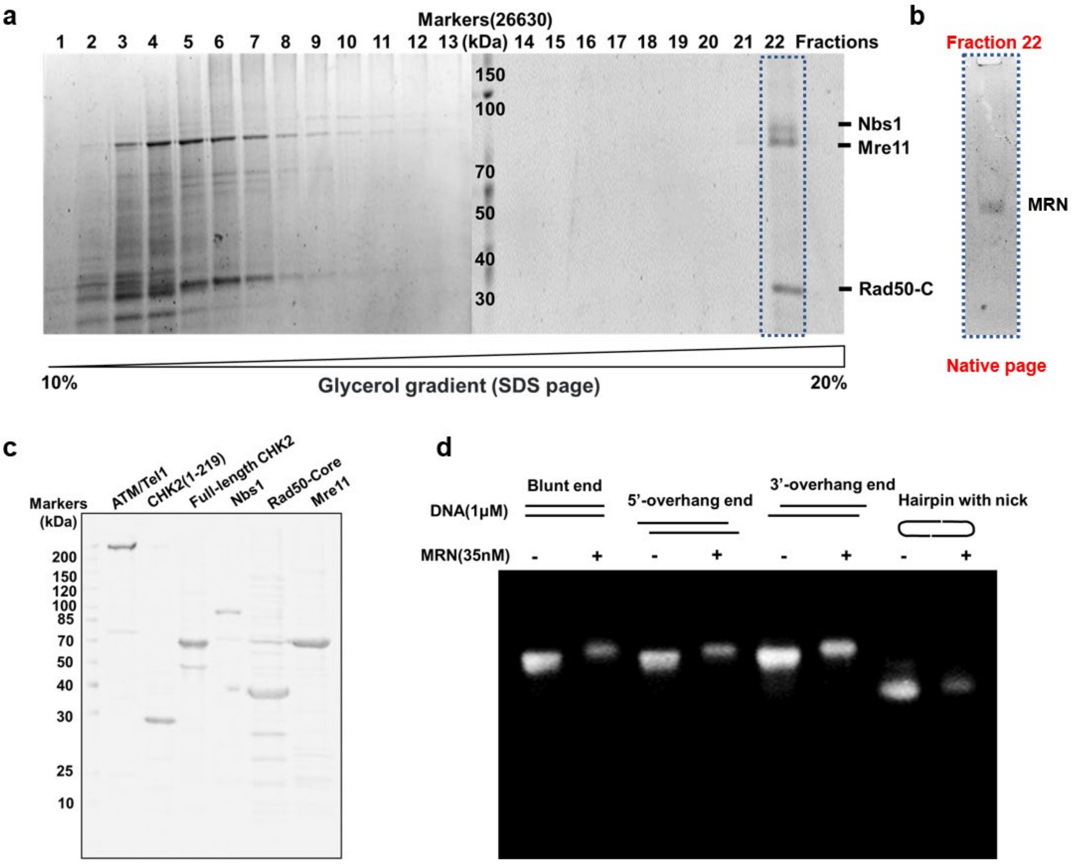

Fig. S4: Functional MRN complex assembled *in vitro* through glycerol gradient centrifugation. (a) SDS–PAGE analysis of the gradient fractions. MRN complex was subjected to 10–20% glycerol gradient centrifugation. Fractions were collected and resolved on SDS–PAGE. (b) Fraction 22 analyzed by native-PAGE shows the formation of the MRN complex. (c) SDS-PAGE analysis of purified MRN, ATM/Tel1, and CHK2 proteins. (d) MRN efficiently cleaves various types of DNA oligonucleotides, demonstrating its nuclease activity.

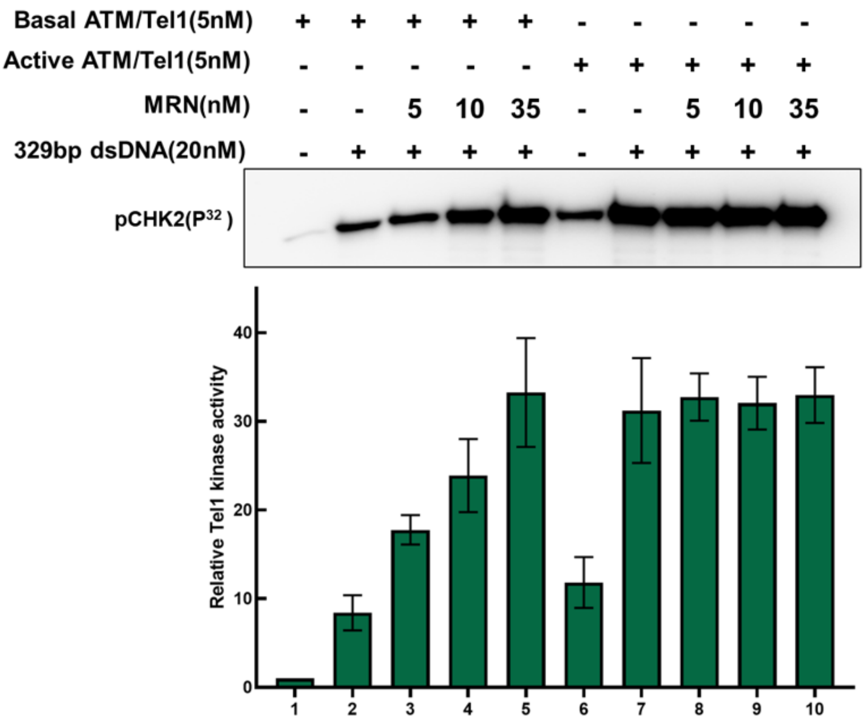

Fig. S5: MRN does not further activate active ATM/Tel1.

(a) *In vitro* kinase assays conducted with both basal and active ATM/Tel1 (5 nM) in the presence of either 20 nM 329 bp blunt-ended DNA fragments or MRN at different concentrations. Bar graphs represent relative kinase activities, quantified from phosphorimaging data. Values shown are averages with standard errors, derived from three independent experiments.

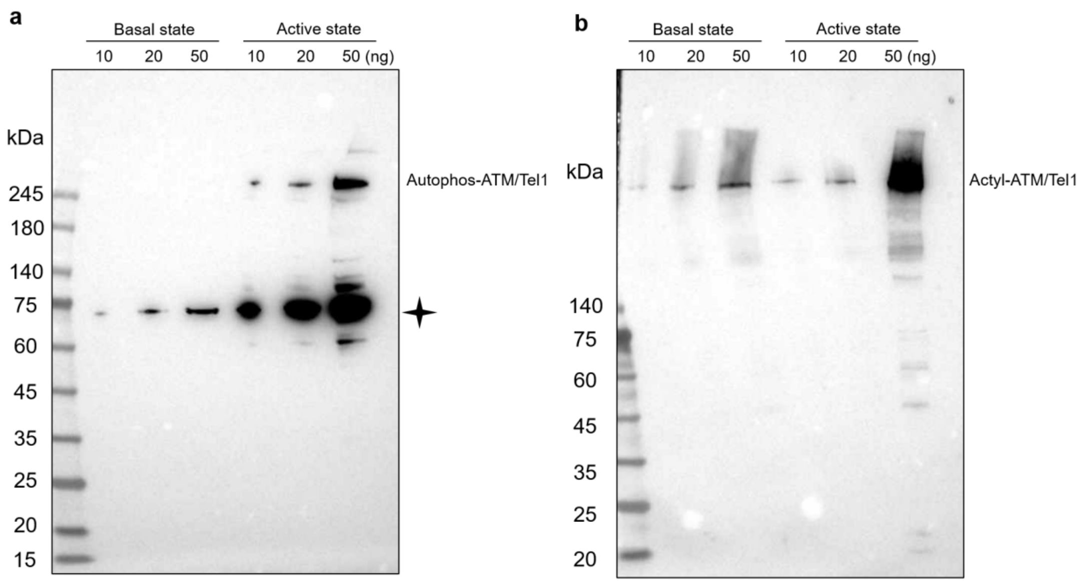

Fig. S6: Active ATM/Tel1 carries multiple post-translational modifications (PTMs).

Purified basal and active ATM/Tel1 were subjected to Western blot (WB) analysis using (a) phospho-ATM/ATR substrate motif (pS/pT)Q and (b) pan-acetylated-lysine antibodies. The specific amounts of ATM/Tel1 used are indicated. An asterisk indicates the phosphorylated bands corresponding to ATM/Tel1 co-purified substrates. The experiment was independently repeated three times.

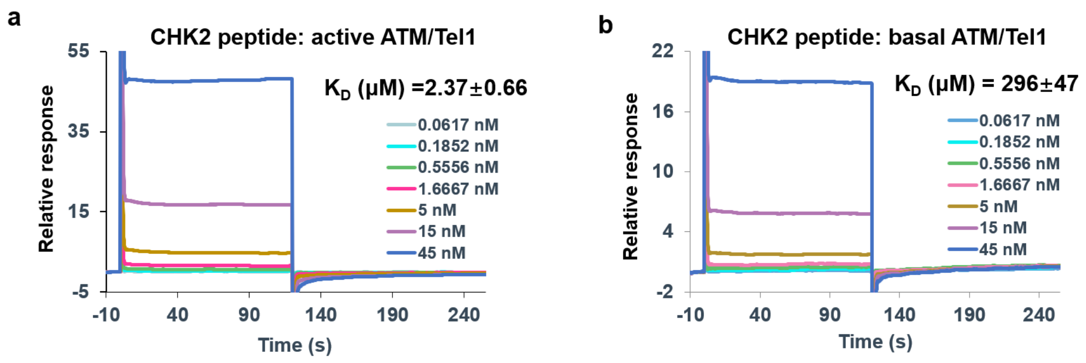

Fig. S7: Surface plasmon resonance (SPR) sensorgram illustrating the binding affinity of the substrate CHK2 peptide to ATM/Tel1 in two distinct states.

(a) The sensorgram shows the binding of the CHK2 peptide with active ATM/Tel1 at varying concentrations of the kinase, reflecting increased affinity in the active state. (b) Conversely, the sensorgram for basal ATM/Tel1 shows significantly lower affinity for the CHK2 peptide, indicating that the kinase's active site is largely inaccessible in the basal state. The experiment was independently repeated three times.

a

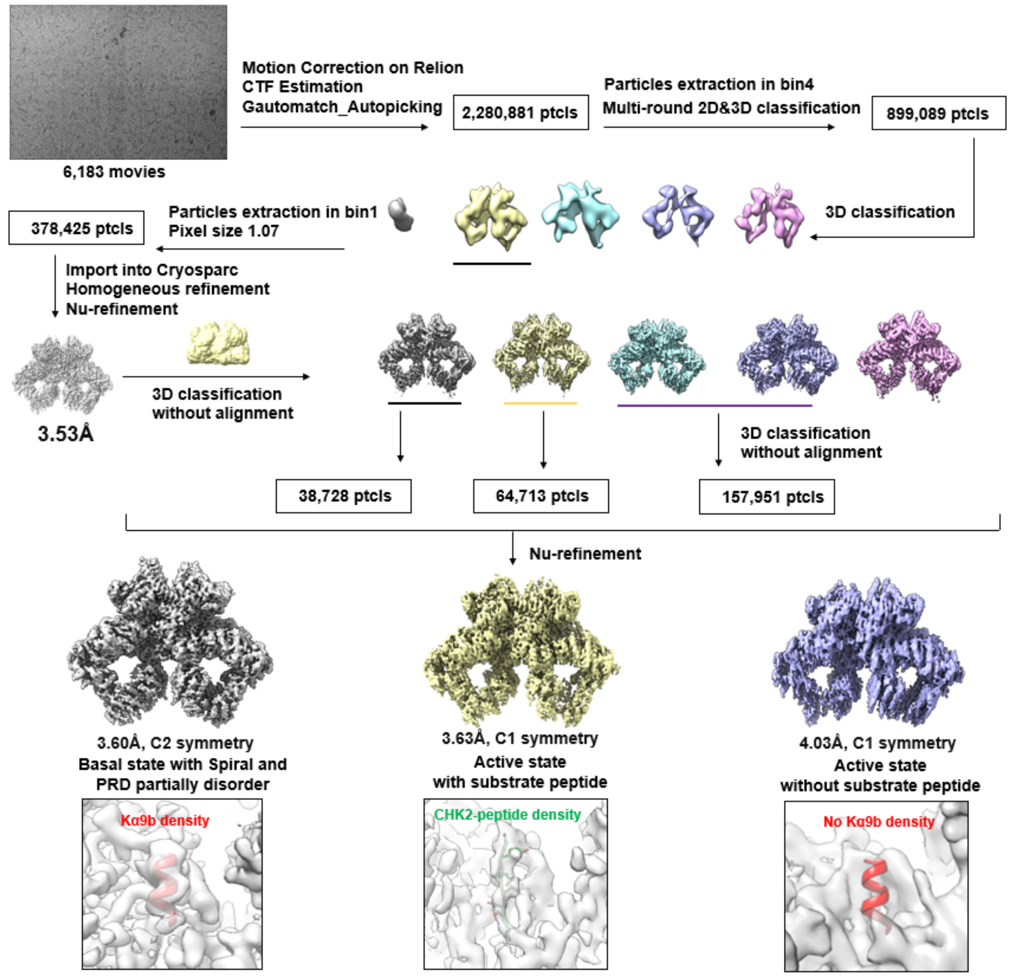

6,183 movies
Motion Correction on Relion
CTF Estimation
Gautomatch_Autopicking
2,280,881 ptcls
Particles extraction in bin4
Multi-round 2D&3D classification
899,089 ptcls
3D classification
Particles extraction in bin1
Pixel size 1.07
378,425 ptcls
Import into Cryosparc
Homogeneous refinement
Nu-refinement
3.53Å
3D classification
without alignment
3D classification
without alignment
38,728 ptcls
64,713 ptcls
157,951 ptcls
Nu-refinement
3.60Å, C2 symmetry
Basal state with Spiral and
PRD partially disorder
3.63Å, C1 symmetry
Active state
with substrate peptide
4.03Å, C1 symmetry
Active state
without substrate peptide
Kα9b density
CHK2-peptide density
No Kα9b density

b

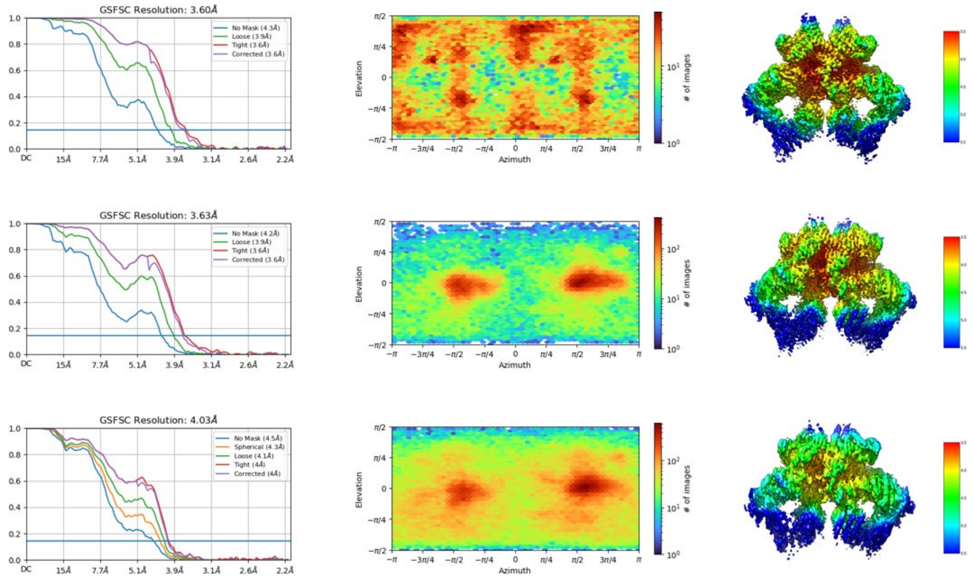

GSFSC Resolution: 3.60Å
GSFSC Resolution: 3.63Å
GSFSC Resolution: 4.03Å
Elevation
Azimuth
# of images

Fig. S8: Cryo-EM reconstruction of ATM/Tel1 in active state.

(a) The schematic diagram illustrates the reconstruction procedures conducted to determine the structure of active ATM/Tel1, revealing three distinct conformations. (b) FSC curve for the cryo-EM density map according to the gold-standard criterion (left). Angular distributions of particles used in the final 3D reconstruction (middle) and the local resolution of the corresponding cryo-EM map (right).

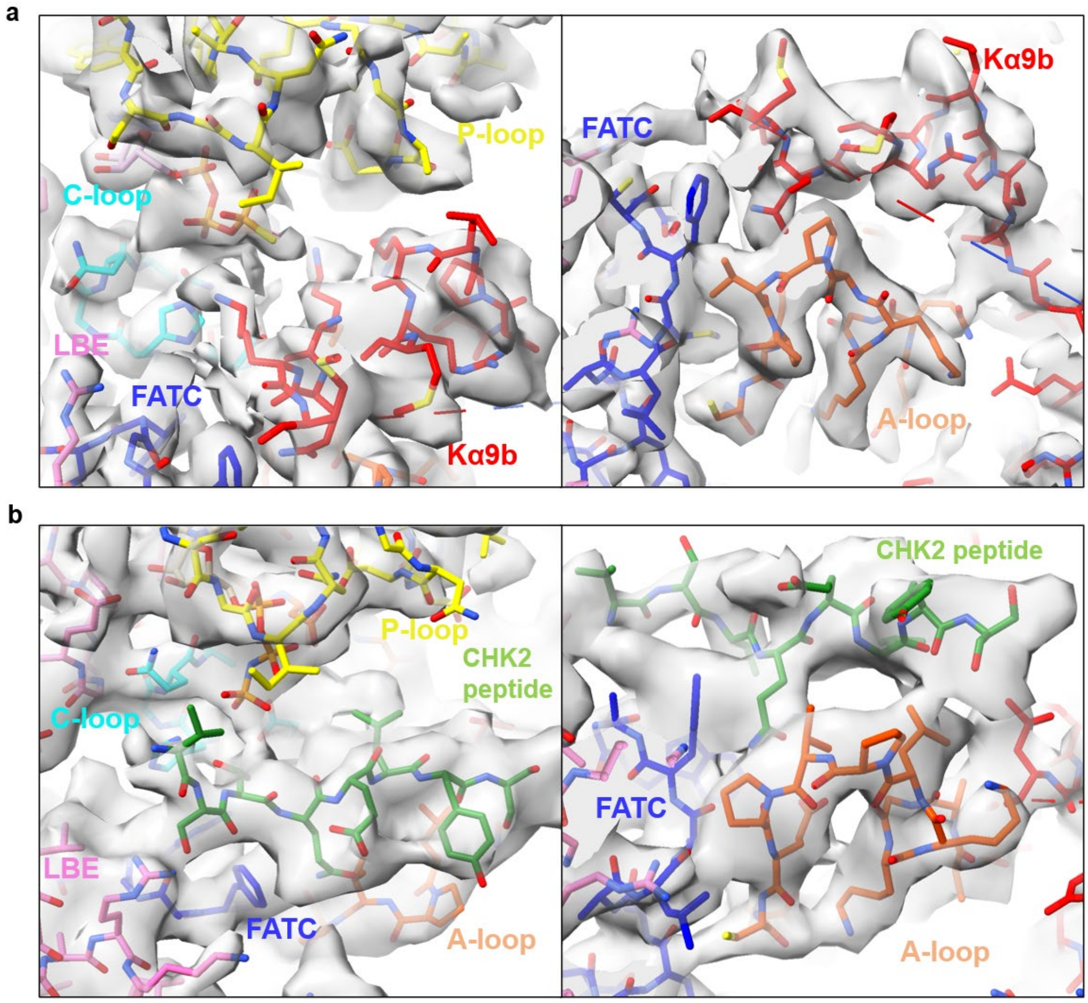

Fig. S9: Model-to-map fitting of the active site of ATM/Tel1 in the active state, shown from different perspectives.

(a) Model-to-map fitting of the active site in Mono2 of ATM/Tel1 in the active state. (b) Model-to-map fitting of the active site in Mono1 of ATM/Tel1 in the active state.

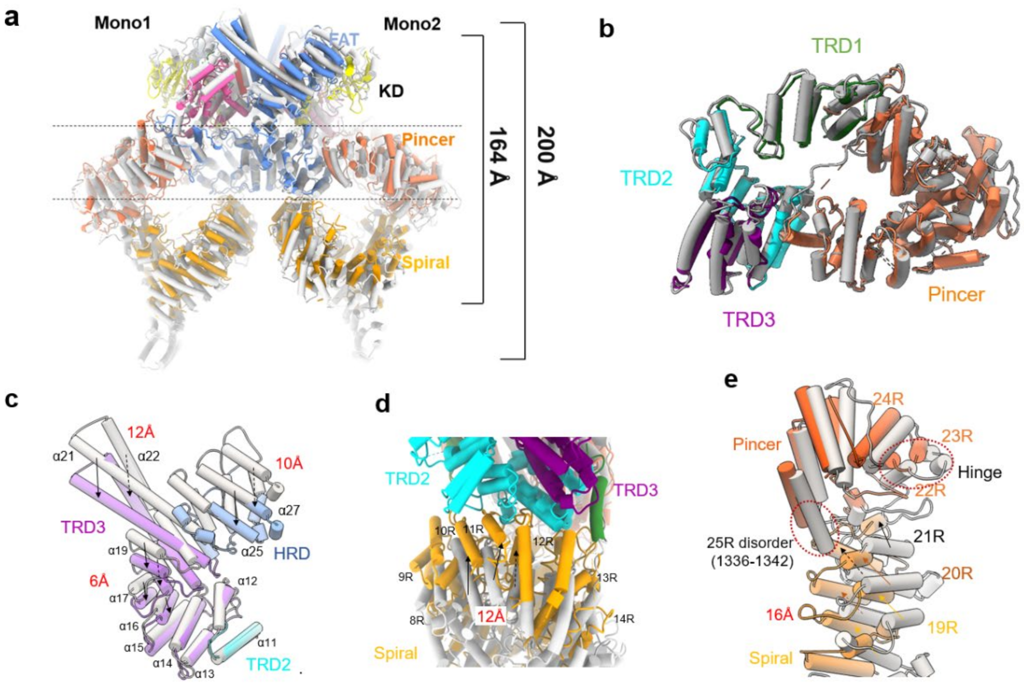

Fig. S10: Transition of ATM/Tel1 from the basal state to the active state.

(a) Structure comparison of the active dimer (domain color-coded as in Fig. 1e) and the basal dimer (gray pipes), and aligned on the Pincer of Mono1 (b) Magnified view of the superimposed Pincer, TRD1, TRD2, and part of TRD3 in the basal state and the active Mono2. (c) Close-up view of the superimposed TRD2, TRD3, and HRD in the basal state and the active Mono2. (d) Close-up view of the superimposed Spiral, TRD2, and TRD3 in the basal state and the active Mono2. (e) Magnified view of the superimposed Spiral and Pincer in the basal state and the active Mono2.

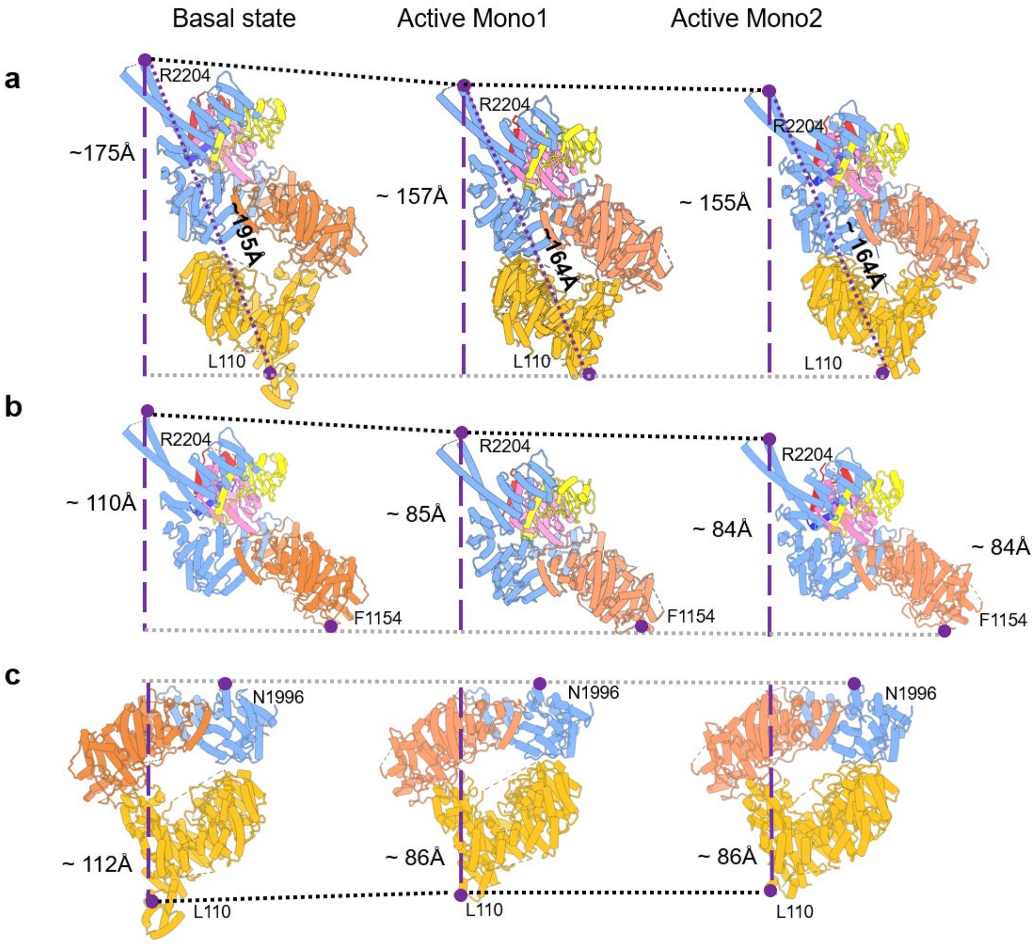

Fig. S11: Conformational differences in ATM/Tel1 between the basal state and the active state. ATM/Tel1 undergoes compaction upon activation. The dashed purple lines indicate the relative heights of each conformer and subunit. (a) Perpendicular distances and straight-line distances between Arg 2204 and Leu 110 (purple dots) are shown below. (b) Perpendicular distances between Arg 2204 and Phe 1154 are shown below. (c) Perpendicular distances between Asn 1996 and Leu 110 are shown below.

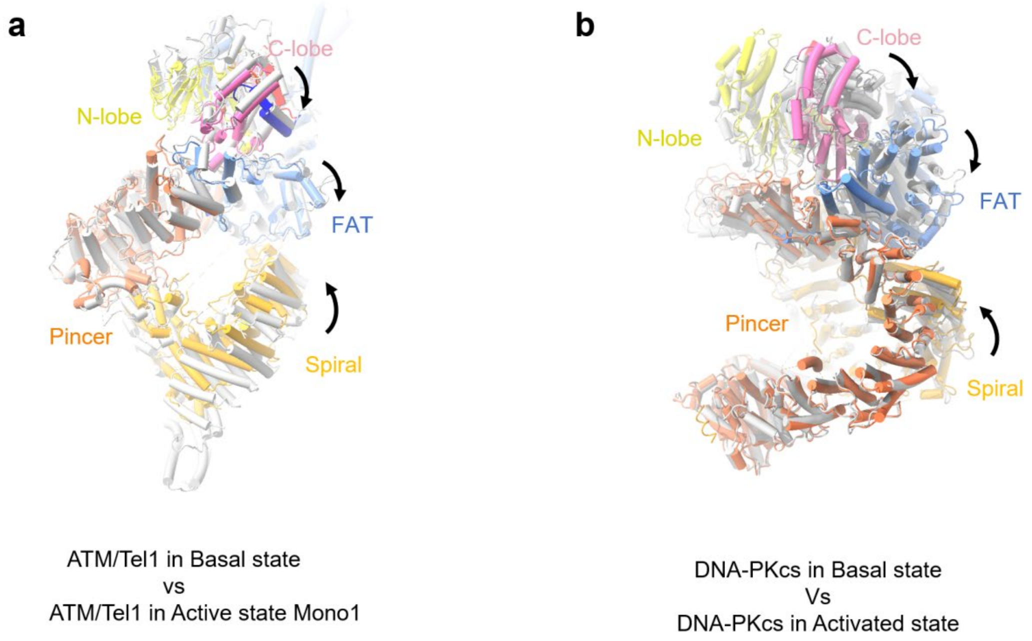

Fig. S12: Comparison of the global changes of ATM/Tel1 and DNA-PKcs required for activation.

(a) shows the superimposed structures of the activated Mono1 (colored) and the basal state (gray pipes). (b) illustrated the superimposed structures of DNA-PKcs of the activated state (PDB: 7k0y, colored) and the basal state (PDB: 7K1J, gray pipes)

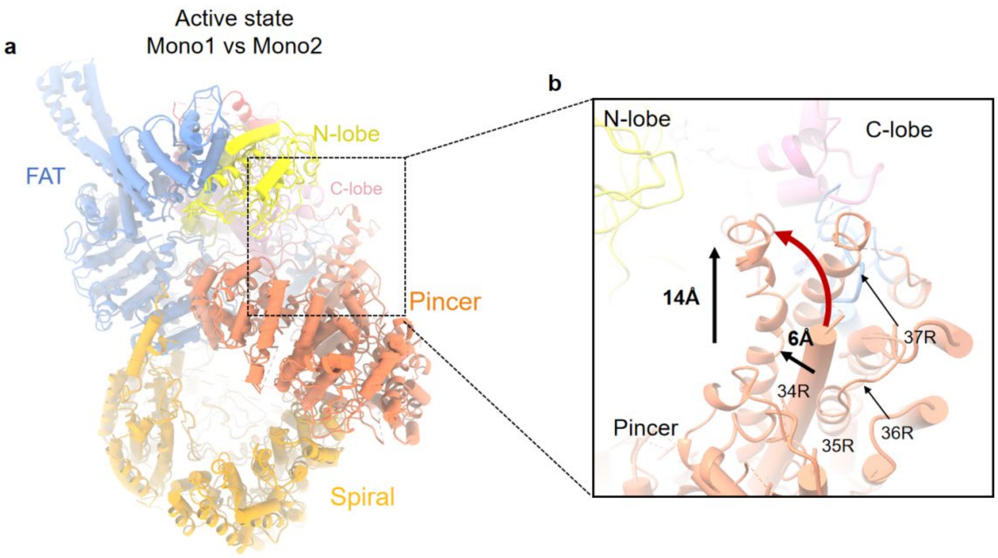

Fig. S13: Close-up views of superimposed of the two active ATM/Tel1 monomers.
(a) Overlay of the structures of active Mono1 (shown as ribbons) and Mono2 (shown in pipes), with their C-lobes (2535-2718) are superimposed. (b) Close-up view of the superimposed Pincer and N-lobe in two monomers, highlighting that the HEAT repeat 34 R of Mono1 moves closer to the N-lobe by 14 Å and 6 Å in two directions, respectively.

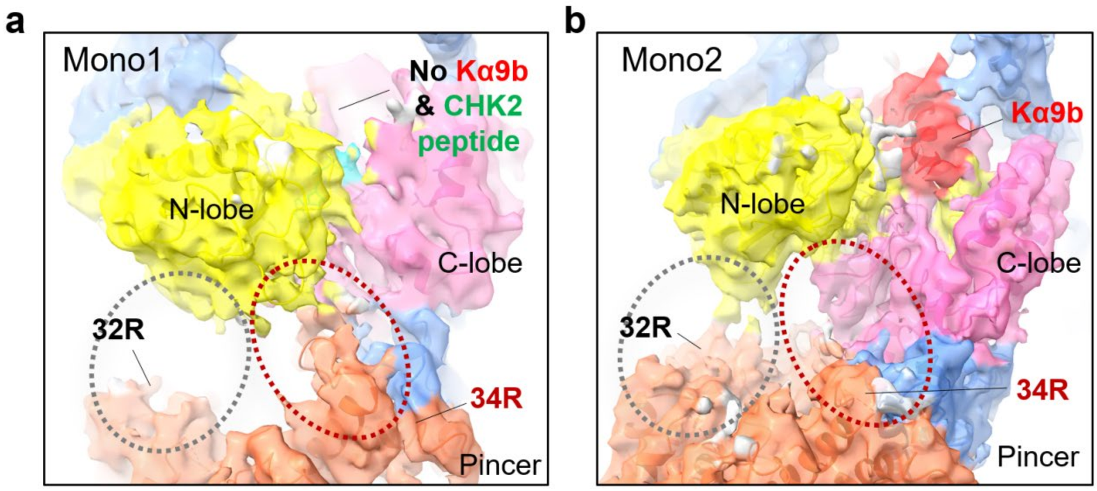

Fig. S14: Structural comparison of the two monomers in active ATM/Tel1 without substrate peptide.

Comparison of the two active monomers highlights the alternate docking of the N-lobe onto distinct regions of the Pincer. This configuration resembles that of ATM/Tel1 bound to the CHK2 peptide. However, in Mono1, neither the PRD kα9b nor the substrate peptide is bound, whereas in Mono2, PRD kα9b occupies the binding site.

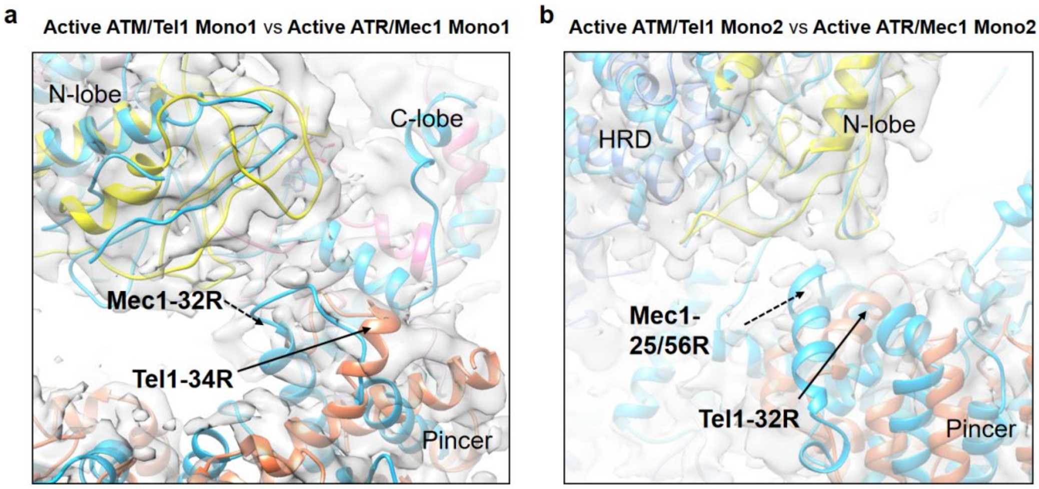

Fig. S15: Superimposed structures of the active ATM/Tel1 and the active ATR/Mec1 disclosing both harbor two alternative N-lobe docking sites.

(a) Highlighting that the HEAT repeat 34 R of active ATM/Tel1 Mono1 corresponds to 32 R of Mono1 of the active ATR/Mec1. (b) Highlighting that the HEAT repeat 32 R of active Mono2 corresponds to 25/26 R of Mono2 of the active ATR.

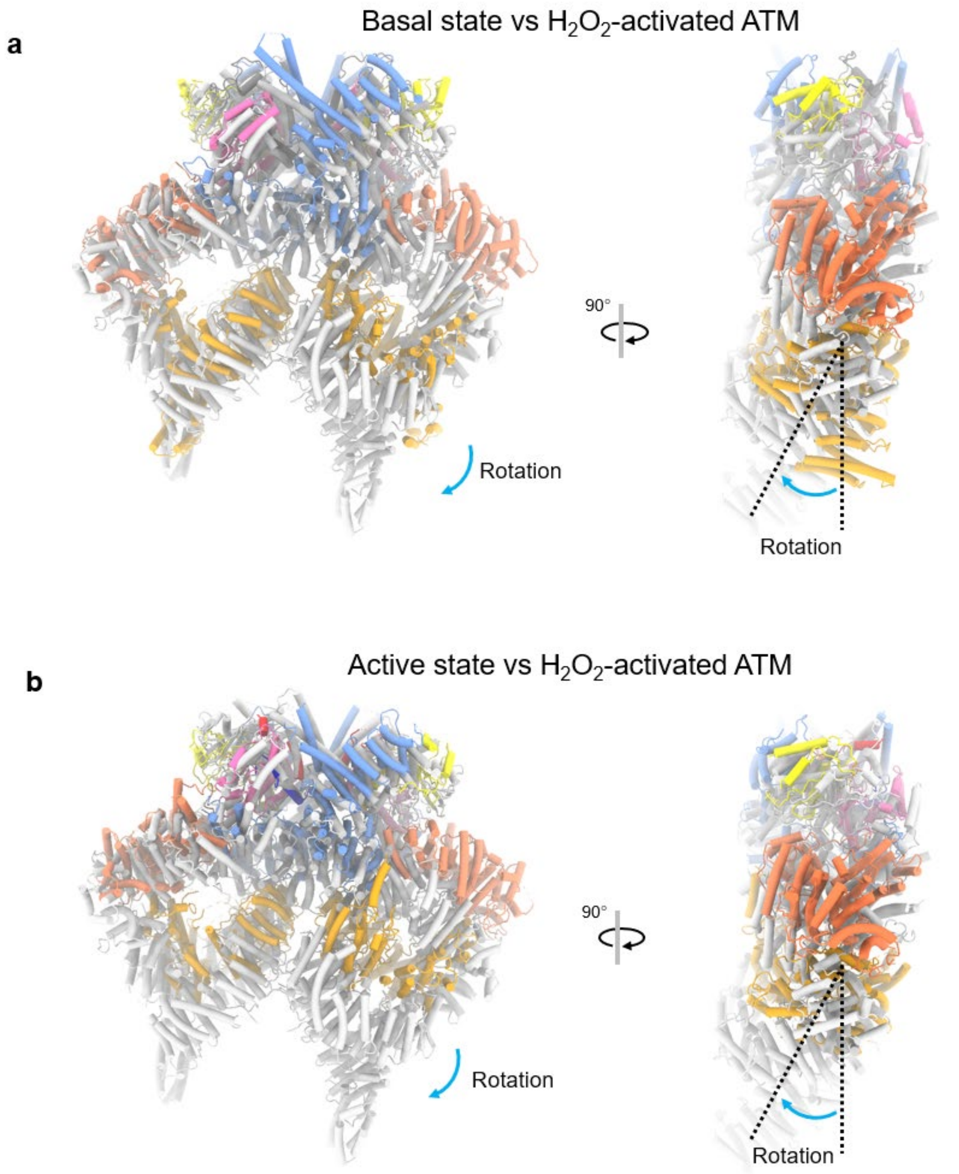

Fig. S16: Comparison of H_2O_2-activated ATM with ATM/Tel1 in the basal and the active states. (a) Comparison of H_2O_2-activated ATM with ATM/Tel1 basal state. (b) Comparison of H_2O_2-activated ATM with ATM/Tel1 active state. The range of motions is indicated.

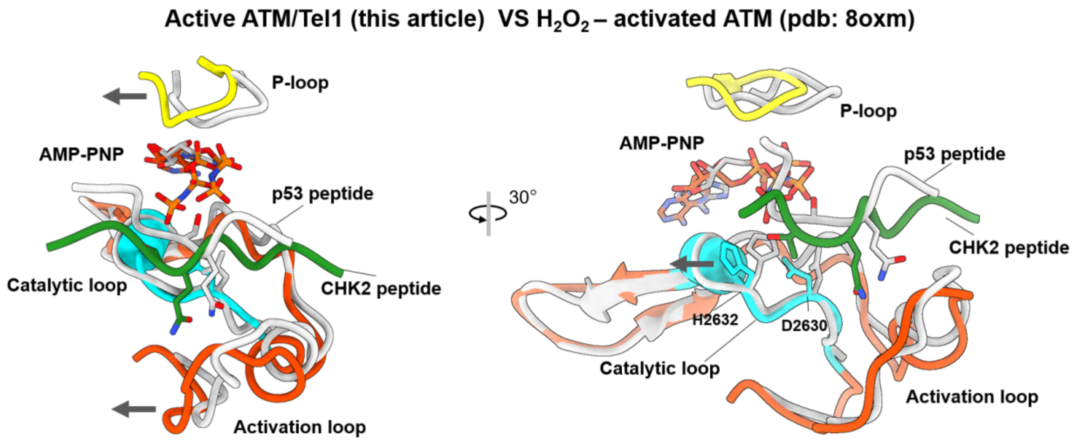

Fig. S17: Comparison of active sites in H_2O_2-activated ATM and active ATM/Tel1.
Comparison of active sites of H_2O_2-activated ATM with ATM/Tel1 in the active state, aligning on the catalytic loop in the kinase C-lobe, highlights differences in the P-loop, activation loop, AMP-PNP binding, and substrate peptide positioning. Two views are shown and are rotated 30° relative to one another.

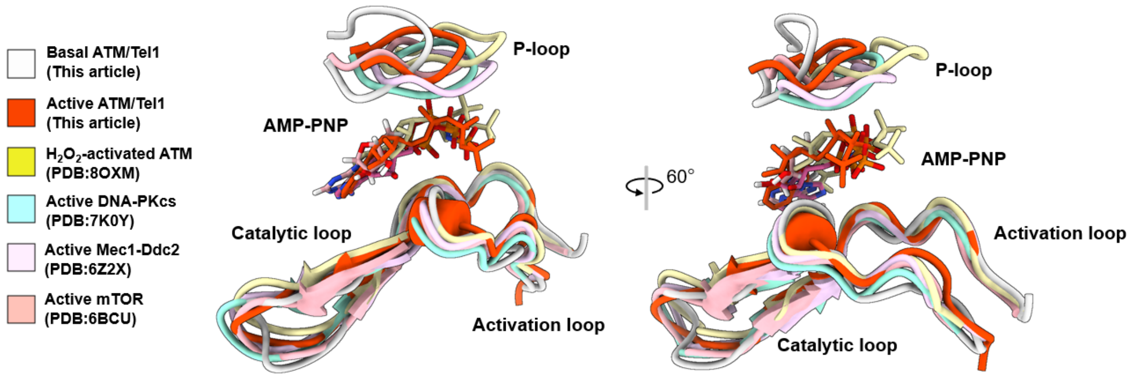

Fig. S18: Comparison of activated PIKK structures with ATM/Tel1 in the basal and the active states.

Activated PIKK structures are superimposed on the ATM/Tel1 basal structure, aligning on the catalytic loop and activation loop in the kinase C-lobe to compare the P-loop shifts in the N-lobe. Two views are shown and are rotated 60° relative to one another.

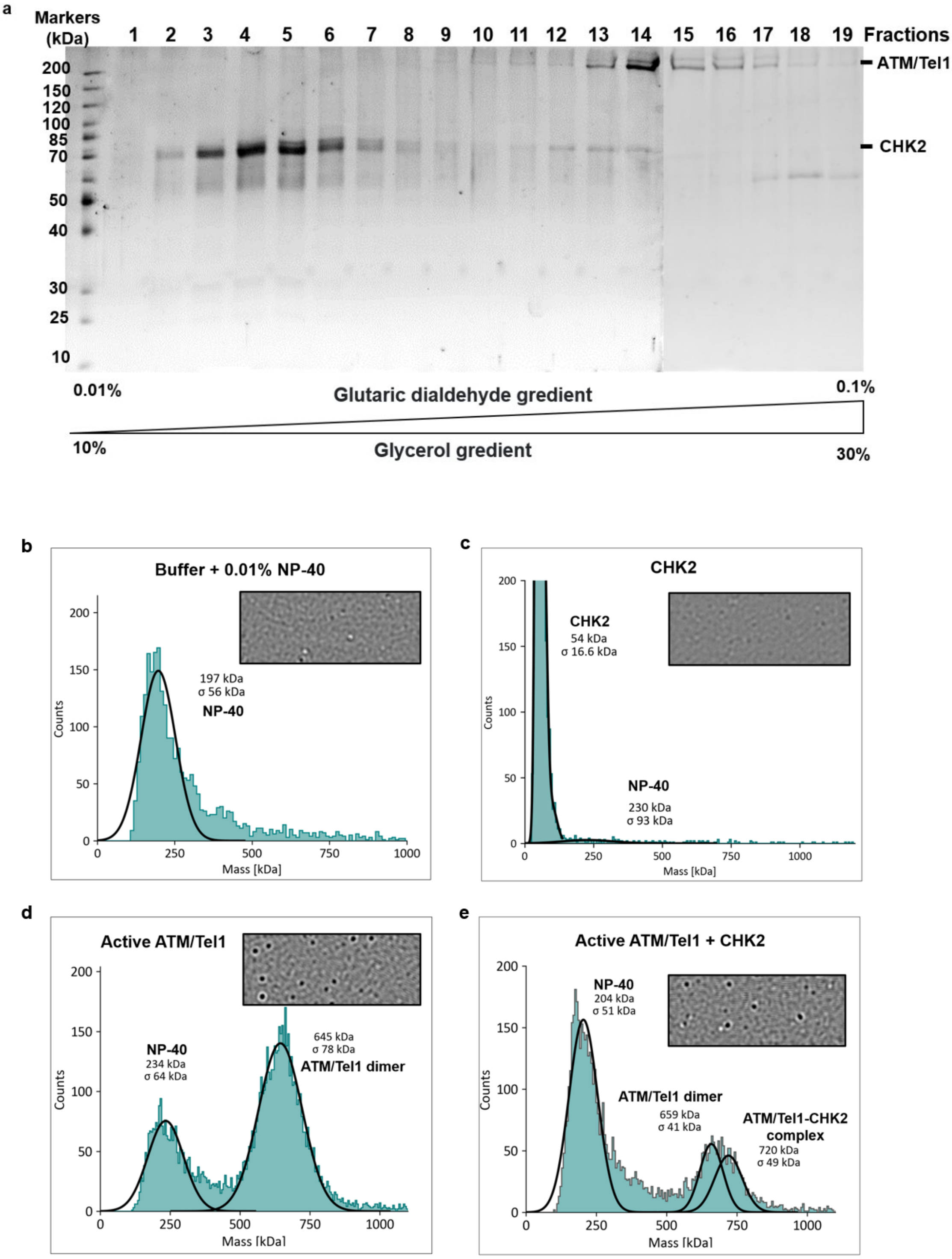

Fig. S19: The active ATM/Tel1 dimer binds only one CHK2 substrate.

(a) Active ATM/Tel1 was incubated with an over-saturated full-length CHK2 complex and fractionated using GraFix. Peak ATM/Tel1-CHK2 complex fractions were combined for further analysis. (b) Mass photometry was used to determine the molecular weight and stoichiometry of the ATM/Tel1-CHK2 complex.

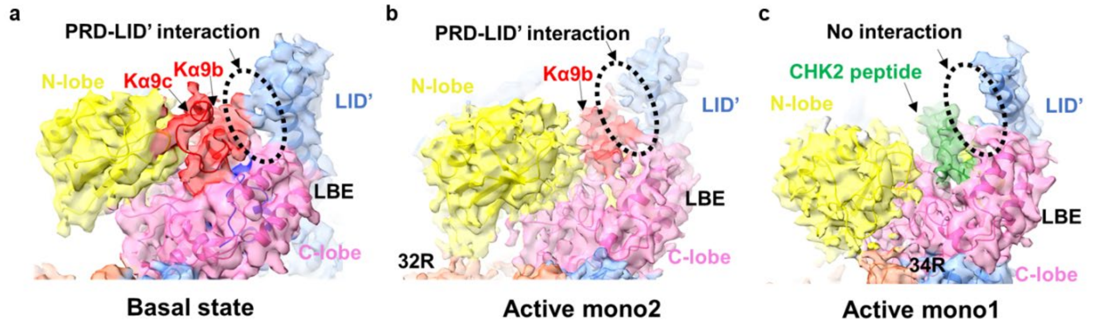

Fig. S20: Conformational differences in the LID-PRD dimer interface of ATM/Tel1 in different functional states.

(a, b) In the basal state and active Mono2, tight interactions between PRD and LID' stabilize Kα9b. (c) In active Mono1, Kα9b becomes disordered and disrupts the PRD-LID' interface, creating space for the substrate to enter the active site.

Table S1. Statistics of 3D reconstruction and model refinement

	**Basal state PDB:9IZ7 (EMD-61024)**	**Active state with substrate peptide PDB:9IZ0 (EMD-61006)**	**Basal state with Spiral and PRD partially disorder (EMD-61018)**	**Active state without substrate peptide (EMD-61014)**
**Date collection and prosessing**				
EM equipment		FEI Titan Krios		
Detector	K2	K3	K3	K3
Magnification	105K	81K	81K	81K
Voltage (kV)	300	300	300	300
Electron exposure (e–/Å^2）	60	50	50	50
Defocus range (μm)	-2.5 to -3.5	-1.5 to -2.5	-1.5 to -2.5	-1.5 to -2.5
Pixel size (Å)		1.07	1.07	1.07
Symmetry imposed	C2	C1	C1	C2
Initial particle images (no.)	6W	228w	228w	228w
Final particle images (no.)	17894	64,713	157,951	38,728
Map resolution (Å)	4.35	3.63	4.03	3.60
FSC threshold	0.143	0.143	0.143	0.143
Map sharpening B factor (A˚ ^{2})	400	107.4	171.3	108.8
**Model composition**				
Nonhydrogen atoms	13031	32117		
Protein residues	2618	4876		
Ligands		2(AMPPNP)		
**R.m.s. deviations**				
Bond lengths (Å)	0.004	0.003		
Bond angles (°)	1.044	0.657		
Clashscore	7	12		
MolProbity score	1.92	2.02		
Ramachandran plot				
Favored (%)	91.13	94.00		
Allowed (%)	8.71	5.74		
Disallowed (%)	0.15	0.19		

Table S2. DNA sequences used for kinase assay

DNA oligos	DNA substrate diagram	DNA sequence (5'-3')
58nt-5'overhang-1	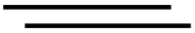	TTTTTGTGCCAGTGCATGTCCGGACTGCTCGTGATCGACA<br>TACAGTTCGACGTCGACA
58nt-5'overhang-2		TTTTTGTCGACGTCGAACTGTATGTCGATCACGAGCAGTC<br>CGGACATGCACTGGCACA
58nt-3'overhang-1	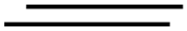	TGTGCCAGTGCATGTCCGGACTGCTCGTGATCGACATACA<br>GTTCGACGTCGACATTTT
58nt-3'overhang-2		TGTCGACGTCGAACTGTATGTCGATCACGAGCAGTCCGGA<br>CATGCACTGGCACATTTT
54nt-blunt-1	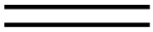	TGTGCCAGTGCATGTCCGGACTGCTCGTGATCGACATACA<br>GTTCGACGTCGACA
54nt-blunt-2		TGTCGACGTCGAACTGTATGTCGATCACGAGCAGTCCGGA<br>CATGCACTGGCACA
54nt-Hairpin	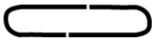	TCAGAAGCAGTAGAGCATGCATATATGCATGCTCTACTGC<br>TTCTGACGATATCG
329bp-blunt	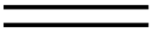	TTCGAGACAGTAATCCGCTCAAAACCTTGGTTCTTTATATA<br>ATGGATATGGCTTCTAAAAACGTATTCATAAAGCCTCAAGA<br>ATTTGATCACGATGAGTATTTCTCCCAGGAGGAAGAAGATA<br>TTTATCGGCCAGAAAATTTAATCAGGAATCATCAAATACTT<br>GGTTTGATGGAAGGATCGCTTGAGCAAATACGGAATACTG<br>ATCTTTTTATTTTACAAAAATATATCGACTACTTTTCTAGTCA<br>TCCTCATGACAGCTTAATTAATATTCTTCATTTGTATCCCAT<br>TGAAACTTTTTGTTTTGGTATGTCTGCAATTGGTGCATAT

Supplementary Reference:

1. Wang, X., et al., *Structure of the intact ATM/Tel1 kinase.* Nature Communications, 2016. **7**(1): p. 11655.https://doi.org/10.1038/ncomms11655.
2. Takagi, Y., et al., *Preponderance of free mediator in the yeast Saccharomyces cerevisiae.* J Biol Chem, 2005. **280**(35): p. 31200-7.https://doi.org/10.1074/jbc.C500150200.
3. Zheng, S.Q., et al., *MotionCor2: anisotropic correction of beam-induced motion for improved cryo-electron microscopy.* Nat Methods, 2017. **14**(4): p. 331-332.https://doi.org/10.1038/nmeth.4193.
4. Rohou, A. and N. Grigorieff, *CTFFIND4: Fast and accurate defocus estimation from electron micrographs.* J Struct Biol, 2015. **192**(2): p. 216-21.https://doi.org/10.1016/j.jsb.2015.08.008.
5. Punjani, A., M.A. Brubaker, and D.J. Fleet, *Building Proteins in a Day: Efficient 3D Molecular Structure Estimation with Electron Cryomicroscopy.* IEEE Trans Pattern Anal Mach Intell, 2017. **39**(4): p. 706-718.https://doi.org/10.1109/tpami.2016.2627573.
6. Jansma, M., et al., *Near-Complete Structure and Model of Tel1ATM from Chaetomium thermophilum Reveals a Robust Autoinhibited ATP State.* Structure, 2020. **28**(1): p. 83-95.e5.https://doi.org/10.1016/j.str.2019.10.013.
7. Bienert, S., et al., *The SWISS-MODEL Repository-new features and functionality.* Nucleic Acids Res, 2017. **45**(D1): p. D313-d319.https://doi.org/10.1093/nar/gkw1132.
8. Pettersen, E.F., et al., *UCSF Chimera--a visualization system for exploratory research and analysis.* J Comput Chem, 2004. **25**(13): p. 1605-12.https://doi.org/10.1002/jcc.20084.
9. Terwilliger, T.C., et al., *Improvement of cryo-EM maps by density modification.* Nat Methods, 2020. **17**(9): p. 923-927.https://doi.org/10.1038/s41592-020-0914-9.
10. Emsley, P. and K. Cowtan, *Coot: model-building tools for molecular graphics.* Acta Crystallogr D Biol Crystallogr, 2004. **60**(Pt 12 Pt 1): p. 2126-32.https://doi.org/10.1107/s0907444904019158.
11. Prisant, M.G., et al., *New tools in MolProbity validation: CaBLAM for CryoEM backbone, UnDowser to rethink "waters," and NGL Viewer to recapture online 3D graphics.* Protein Sci, 2020. **29**(1): p. 315-329.https://doi.org/10.1002/pro.3786.
12. Goddard, T.D., et al., *UCSF ChimeraX: Meeting modern challenges in visualization and analysis.* Protein Science, 2018. **27**(1): p. 14-25.https://doi.org/https://doi.org/10.1002/pro.3235.
